# Assessing the Utility of Predicted Brain Age for Explaining Variability in Language Abilities in Healthy Older Adults

**DOI:** 10.1162/NOL.a.21

**Published:** 2025-11-03

**Authors:** Yanina Prystauka, Foyzul Rahman, Natalie Busby, Jens Roeser, Carl-Johan Boraxbekk, Jack Feron, Samuel J. E. Lucas, Allison Wetterlin, Eunice G. Fernandes, Linda Wheeldon, Katrien Segaert

**Affiliations:** Department of Linguistic, Literary and Aesthetic Studies, University of Bergen, Bergen, Norway; School of Sport, Exercise, and Health Sciences, Loughborough University, Loughborough, UK; Department of Communication Sciences and Disorders, University of South Carolina, Columbia, SC, USA; Department of Psychology, Nottingham Trent University, Nottingham, UK; Institute for Clinical Medicine, Faculty of Medical and Health Sciences, University of Copenhagen, Copenhagen, Denmark; Institute of Sports Medicine Copenhagen (ISMC) and Department of Neurology, Copenhagen University Hospital Bispebjerg, Copenhagen, Denmark; School of Sport, Exercise and Rehabilitation Sciences, University of Birmingham, Birmingham, UK; Centre for Human Brain Health, University of Birmingham, Birmingham, UK; Department of Foreign Languages and Translation, University of Agder, Kristiansand, Norway; School of Psychology, University of Minho, Braga, Portugal; School of Psychology, University of Birmingham, Birmingham, UK

**Keywords:** ageing, brain age, brain structure, comprehension, production

## Abstract

We investigated whether the difference between chronological and modeled brain age explains individual differences in language performance among healthy older adults. Age-related decline in language abilities is widely documented, with considerable variability among healthy older individuals in both language performance and underlying neural substrate. We derived predicted brain age from grey and white matter using machine learning and used this measure to estimate neurological deviations from chronological age. Using Bayesian mixed-effects modeling, we tested whether brain-age deviations predict language performance in a sample of 86 adults aged 60 years and above. We assessed the effect of brain-age deviations on performance across four well-established language processing tasks, each tapping into linguistic domains known to be vulnerable to ageing and show individual variability in skill levels, in both comprehension and production. Our findings suggest that, in healthy older individuals, predicted deviations of brain age from chronological age do not predict language abilities. This challenges the idea that brain age is a reliable determinant of language processing variability, at least in healthy (as opposed to pathological) ageing and highlights the need to consider other neural and cognitive factors when studying language decline.

## INTRODUCTION

The way we process language undergoes changes in older adulthood, yielding a complex picture of preservation in some language functions alongside decline in others. In language production, word-finding difficulties, rooted in the weakening of connections between lexical and phonological representations, are a well-documented hallmark of age-related changes (Burke et al., 1991, 2004; Burke & Shafto, 2008; Gollan & Brown, 2006; Heine et al., 1999; Rossi & Diaz, 2016; Salthouse & Mandell, 2013; Segaert et al., 2018). Another age-related deficit in production is reduced syntactic complexity of spoken and written language, such as fewer embedded clauses and a lower number of clauses per utterance (Kemper, Greiner, et al., 2001; Kemper et al., 2003; Kemper & Sumner, 2001; Kemper, Thompson, & Marquis, 2001). However, some real time production measures suggest that syntactic processing remains relatively preserved, at least for relatively simple structures (Hardy et al., 2020). Ageing also affects language comprehension, although findings vary depending on the tasks and methods used to assess this relationship. Some studies suggest that older adults rely more on contextual information for comprehension than younger adults (Fernandes, Segaert, et al., 2025; Madden, 1988; Pichora-Fuller et al., 1995; Sommers & Danielson, 1999; Steen-Baker et al., 2017; Stine-Morrow et al., 2008). However, work on predictive processing using electroencephalography (Federmeier & Kutas, 2005; Federmeier et al., 2002, 2003) indicates that, compared to younger adults, older adults are slower and less effective at using information from more predictive contexts to guide their word processing. When testing syntactic processing, age-related effects are most often detected in comprehension accuracy rather than processing speed (Caplan & Waters, 2005; DeDe et al., 2004; Obler et al., 1991; Waters & Caplan, 2001; though see also Caplan et al., 2011). Some of the discrepancies in the available literature could stem from the different nature of the linguistic tasks and underlying processes they tap into, as well as from significant variability among individuals in the extent to which age-related decline in these language functions transpires, with some individuals clearly experiencing more decline than others (Federmeier & Kutas, 2005; Federmeier et al., 2010). Chronological age may not fully capture the variability in cognitive processes across individuals, and further research using measures with greater predictive validity is needed. One such measure is predicted brain age—a neuroimaging-based marker that indicates whether a person’s brain appears older or younger than average for their chronological age—which may more closely reflect biological ageing. In this article, we examine the potential of predicted brain age as a determinant of individual differences in language abilities.

Previous research links age-related changes in cognitive (including language) functions to the degeneration of brain matter (Charlton et al., 2006; Ferreira et al., 2014; Koini et al., 2018; Lockhart et al., 2012; Oschwald et al., 2019). There is a general pattern of age-related brain atrophy, with different regions of grey and white matter undergoing distinct trajectories of change in both pace and extent (Fjell & Walhovd, 2010; Hedman et al., 2012). Despite extensive ongoing research aimed at identifying the neural structural correlates of changes in linguistic function during healthy ageing (Diaz et al., 2016; Houston et al., 2019; Oschwald et al., 2019; Pelletier et al., 2017; Rizio & Diaz, 2016; Shafto et al., 2007; Stamatakis et al., 2011; Zhang et al., 2013; Zhu et al., 2022), it is still not possible to draw definitive conclusions about the relationship between brain structural changes and language performance in this context. One of the challenges lies in isolating language-specific processes from domain-general cognitive functions, as linguistic processing typically engages multiple neural systems, including those involved in attention, memory, and executive function. There are discussions in the literature regarding whether each of these functions is independently affected by ageing or whether there is a global developmental process, underlying changes in these different domains (Tucker-Drob, 2011). This overlap introduces an additional layer of complexity to efforts aimed at mapping the relationship between brain structure and language in ageing. Another challenge is substantial individual variability (Raz et al., 2005, 2010): unlike chronological ageing, biological ageing can be modulated by lifestyle factors such as education (Steffener et al., 2016), physical activity (Dunås et al., 2021; Steffener et al., 2016), body mass index (Ho et al., 2011), socioeconomic status (Busby et al., 2023), sleep (Baril et al., 2021), and substance use (Cole, 2020), as well as genetic predispositions (Ferrucci et al., 2020; López-Otín et al., 2013; see also Cabeza et al., 2018; Fratiglioni et al., 2020; Livingston et al., 2020, 2024; Wittens et al., 2024).

In this article, we use a machine learning approach to address the possibility that variability in language abilities is related to whether a person’s brain appears younger or older than expected for their chronological age. Previous research on predicted brain age (described in detail below) has demonstrated that it may account for decline in cognitive performance beyond what would be expected from chronological age (Cole et al., 2018; Dunås et al., 2021; Elliott et al., 2021), providing evidence that brain age prediction could potentially explain variability in ageing whether it is pathological or not (Franke & Gaser, 2012; Liem et al., 2017; Wittens et al., 2024). However, no research to date has investigated if and how predicted brain age relates to specific aspects of language comprehension and production in healthy ageing.

Cole and Franke (2017) describe the process of predicting brain age as follows. First, neuroimaging data, typically T1-weighted structural magnetic resonance imaging (MRI) scans from healthy individuals, are labeled with participants’ chronological ages and used as input for a machine learning regression model. To validate the model’s accuracy, a portion of the participants’ images is excluded during training. For instance, in tenfold cross-validation, the model is trained on 90% of the participants’ data, and then age predictions are generated for the remaining 10%. This process is repeated until predictions have been made for all participants. The predicted values are then compared to the actual chronological ages to evaluate the model’s accuracy. Once the model is deemed sufficiently accurate, it is trained on the entire training set. The resulting model coefficients are then applied to the structural brain scans of new participants to generate brain age predictions. The predicted brain age is then compared with the chronological age of the participants. Brains that appear older—relative to chronological age—are assumed to reflect advanced brain ageing, while those that appear younger suggest slower or healthier brain ageing. The difference between brain age and chronological age can then be analyzed in relation to other participant characteristics.

Associations between brain age and cognitive functioning appear consistent across the literature. However, existing studies primarily rely on broad cognitive assessments and show that the relationship is most robust in pathological samples. For example, Cole et al. (2018) and Elliott et al. (2021) demonstrated that increased brain age was associated with poorer cognitive performance in 45- and 70-year-old individuals. Dunås et al. (2021) found similar evidence for nonpathological ageing. Other studies have focused on the predictive validity of brain age in cohorts exhibiting pathological ageing. For instance, Liem et al. (2017) revealed that more severe objective cognitive impairment was associated with higher brain age scores. Franke and Gaser (2012) report a longitudinal investigation of four groups of participants classified as (1) healthy ageing, or those with (2) stable mild or (3) progressive cognitive impairment, or people with (4) Alzheimer’s disease. They found that brain age scores were moderately correlated with cognitive functioning and clinical disease severity over 4 years. Similarly, Wittens et al. (2024) found an association between overall cognitive impairment (assessed using Mini Mental State Examination scores; Folstein et al., 1975) and brain predicted age difference, primarily observed in individuals with mild cognitive impairment and Alzheimer’s disease, indicating that disease stage may drive this relationship.

It is yet unclear whether and how brain age relates to more nuanced cognitive functions, specifically language. Indeed, existing evidence relating to language abilities is sparse. For example, Kristinsson et al. (2022) tested if brain age at stroke onset is associated with cross-sectional language function and long-term recovery (2.4–5.4 yr post-stroke). Brain age difference significantly accounted for variance in overall language score at stroke onset and a few years later, as well as in naming and speech repetition (BEST-2; West et al., 1998) at stroke onset, beyond chronological age. This study thus demonstrates the effectiveness of a brain age measure in accounting for differences in language outcomes, at least in a population of recovering stroke patients. The only other study that took a psycholinguistic approach to exploring the relationship between brain age and language was presented by Matchin (2023) at the Society for the Neurobiology of Language 15th Annual Meeting. Matchin reported that brain age predicts sentence processing declines in healthy ageing beyond chronological age and working memory. The outcome variable in this work was reading times on subject- and object-relative clauses. However, this effect did not survive more rigorous statistical modeling, and the research group proceeded to investigate other metrics such as grey matter volume which did show age-related effects in language processing (W. Matchin, personal communication, 12 December 2024).

**Figure 1.**
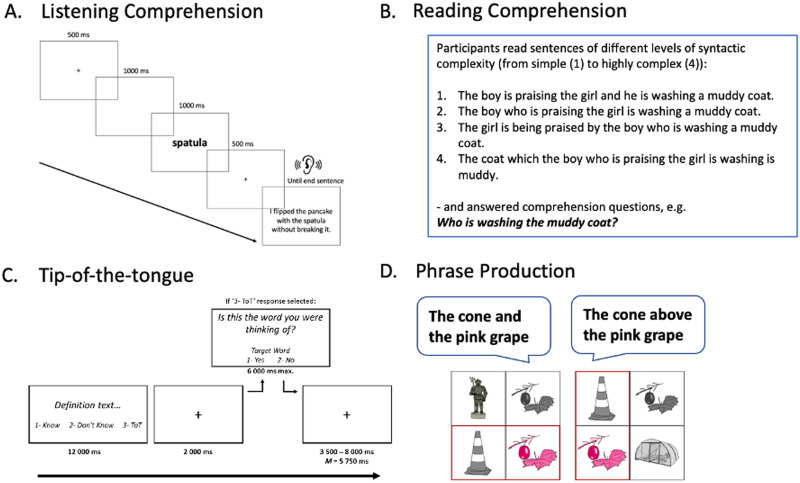
Examples of all four language tasks. (A) Listening comprehension. (B) Reading comprehension. (C) Tip-of-the-tongue (ToT). (D) Phrase production tasks. Each panel illustrates either a single trial, a selection of trials, or an overview of conditions.

As the field of the neurobiology of language is increasingly moving towards explaining variability in language (Kidd et al., 2018; Rothman et al., 2023) among individuals and across the life-span, it is becoming more important to find new approaches to explain such variability. Uncovering the effects of different lifestyle and genetic factors and their interactions on language in ageing is an incredibly complex task. Using machine learning to compute a measure of structural brain health (i.e., predicted brain age) that is sensitive to all these factors and could explain language effects in ageing would be a significant advancement for both the basic and clinical cognitive neuroscience of language. The work on brain age and its relationship with cognition reviewed above does suggest that this is a promising avenue. In the present study, we assessed four key components of language processing, each previously demonstrated to be subject to age-related differences to varying degrees, and investigated whether brain-age gap explained individuals’ performance levels, namely in phonological, lexical, semantic, and syntactic aspects of both comprehension and production processes. We used two sentence comprehension tasks (a listening comprehension task manipulating sentence-level syntax and semantics and a reading comprehension task manipulating levels of syntactic complexity) and two production tasks (a tip-of-the-tongue task to measure single-word retrieval and a phrase production task to measure syntactic production). An overview of the tasks with example stimuli is provided in Figure 1 and Table 1. We hypothesized that larger brain-age gap scores—where brain age appears older relative to chronological age—would be associated with poorer performance across language tasks, particularly in production, which is the hallmark of language processing changes in ageing (Burke et al., 1991, 2004; Rossi & Diaz, 2016; Salthouse & Mandell, 2013; Segaert et al., 2018).

**Table 1.** Conditions and example stimuli across tasks in the study

**Task**	**Conditions**	**Examples**
Listening comprehension	Random word order	Tried I find to quickly the *spatula* without pancake it to flip the breaking.
	Low-constraining (syntactic structure, but no semantic prediction)	I tried to quickly find the *spatula* to flip the pancake without breaking it.
	High-constraining (syntactic structure and semantic prediction)	I flipped the pancake with the *spatula* without breaking it.
Reading comprehension	Simple syntactic structure	The boy is blessing the girl and he is hugging a fuzzy cushion.
	Moderate syntactic structure	The boy who is blessing the girl is hugging a fuzzy cushion.
	Complex syntactic structure	The girl is being blessed by the boy who is hugging a fuzzy cushion.
	Highly complex syntactic structure	The cushion which the boy who is blessing the girl is hugging is fuzzy.
Tip-of-the-tongue	Definition	The act of refusing to cast one’s vote (target word: abstention).
Phrase production	Coordinate simple	The cone and the grape.
	Coordinate complex	The cone and the pink grape.
	Prepositional simple	The cone above the grape.
	Prepositional complex	The cone above the pink grape.

## METHODS

### Participants

The data for the present study were collected as part of a larger study (preregistration: https://osf.io/6fqg7; materials and data for the present report: https://github.com/yanina-prystauka/FAB_BrainAge). Research Question 3 in the preregistration pertains to language performance related to age and we expand upon this by exploring the relationship to brain age. The present contribution has different outcome measures from other publications within the larger project (Fernandes, Fosstveit, et al., 2025; Fernandes, Segaert, et al., 2025; Feron, Rahman, et al., 2024; Feron, Segaert, et al., 2024; Fosstveit et al., 2024; Markiewicz et al., 2025; Rahman et al., 2025). The data in the present contribution are from a subgroup of a larger participant cohort and focuses on those participants for whom T1-weighted structural imaging data, language data and the relevant demographic data were available.

Specifically, 86 participants underwent structural MRI, used to compute predicted brain age. Their chronological age ranged from 60 to 81 years old (mean = 65.5, *SD* = 4.8, *N* females = 42). Due to missing behavioural or demographic data for some participants for some of the tasks, the number of participants included in each task ranged from 80 to 85. We used chronological age, education level, and a measure of working memory (digit span) as background variables in our statistical models. This information is summarized in Table 2 and described in Additional Measures.

**Table 2.** Demographic characteristics of participants

**Measure**	**Value**
Mean age (*SD*)	65.5 (4.8) (range = 60–81)
Mean digit span (*SD*)	5.3 (1.3)
Education	
No formal education	1 (1.2%)
Compulsory	22 (25.6%)
Further	28 (32.6%)
Undergrad	17 (19.8%)
Postgrad	14 (16.3%)
Higher	4 (4.7%)
Male/female	Males *N* = 44; females *N* = 42
Education (yr)	13.7 (2.8)
BMI	27.1 (3.6)
MoCA	27.5 (1.8) (range 23–30)
Sedentariness (mins/day)	631.9 (74)
LPA (mins/day)	174 (39.3)
MVPA (mins/day)	45.4 (22.3)
Parental education	
Compulsory	63 (73.3%)
Further	13 (15.1%)
Undergrad	1 (1.2%)
Postgrad	7 (8.1%)
Parental occupation	
Professional	22 (25.6%)
Intermediate	22 (25.6%)
Manual	39 (45.3%)

*Note*. Inclusion criteria for participation in the study required the absence of hearing loss, a history of concussion or neurological disorders, and any diagnosed learning disabilities. Participants were required to have controlled blood pressure (including those on medication) to be included in our study. Individuals with a diagnosis of diabetes or heart disease were excluded. While cerebrovascular disease and hyperlipidemia were not explicitly screened for, the overall health-related inclusion criteria, along with medication records, suggest that participants with clinically significant conditions were unlikely to be included in the final sample. Sedentariness, LPA and MVPA measures are obtained from accelerometers. BMI = body mass index, MoCA = Montreal Cognitive Assessment, LPA = light physical activity, MVPA = moderate-to-vigorous physical activity.

All participants underwent the Montreal Cognitive Assessment (MoCA; Nasreddine et al., 2005). Only participants who scored 23 or higher on the MoCA (Carson et al., 2018) were included in the study. While the original MoCA study recommended a cutoff of 26 (Nasreddine et al., 2005), later research has shown that this threshold may produce a higher rate of false positives. Carson et al. (2018) found that a cutoff of 23 provides better classification accuracy and is therefore more appropriate for identifying cognitive impairment in older adults. All participants provided informed consent and were compensated for their time. All were British-English monolinguals with no history of speech, language, or other health disorders. The study was granted institutional ethics approval (University of Birmingham, ERN 20_1107) and complied with the Declaration of Helsinki.

### MRI Data Acquisition

The neuroimaging data were acquired using a 3-T Siemens PRISMA system with a 32-channel head-coil at the Centre for Human Brain Health at the University of Birmingham, UK. A T1-weighted 3D-structural MRI (GRAPPA) was acquired with the following parameters: repetition time (TR) = 2,000 ms, echo time (TE) = 2.01 ms, inversion time (TI) = 880 ms, flip angle = 8°, field of view (FOV) = 256 × 256 × 208 mm, voxel dimension (resolution) = 1 mm isotropic, GRAPPA factor = 2; with a total acquisition time of 4 min and 54 s.

### Brain Age Prediction

**Figure 2.**
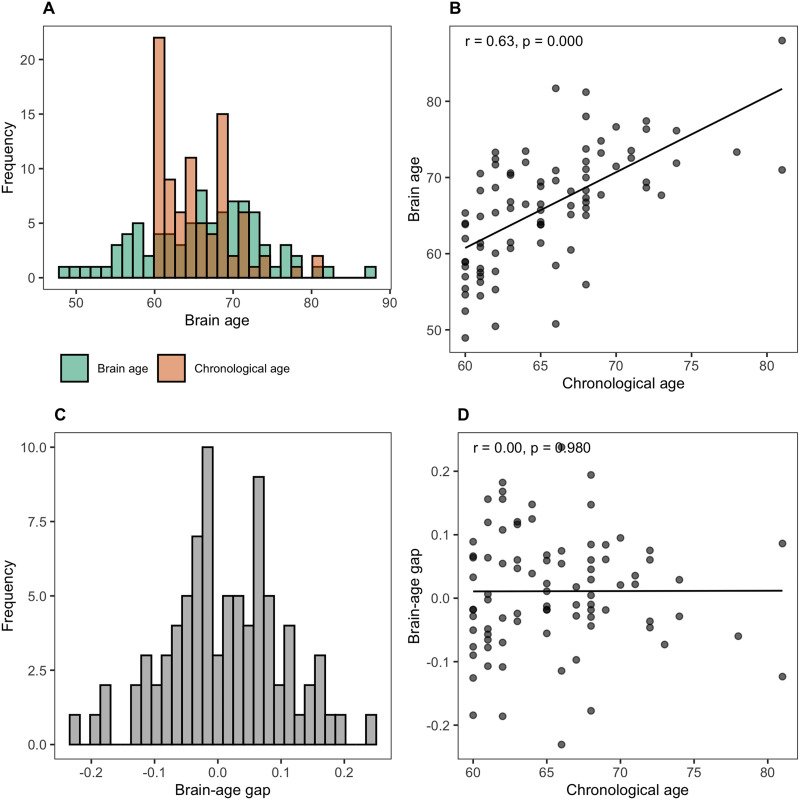
Relationships among chronological age, brain age, and the brain-age gap. (A) Distribution of chronological age and brain age, illustrating that chronological age has a narrower distribution than brain age. (B) Their positive correlation. (C) Distribution of the normalized brain-age gap computed as (brain age − chronological age)/chronological age. (D) Correlation of brain age and chronological age disappears for the normalized brain-age gap scores.

The brainageR model for v2.1 was trained on *n* = 3,377 healthy individuals (mean age = 40.6 years, *SD* = 21.4, age range 18–92 yr) from seven publicly available datasets and tested on *n* = 857 (mean age = 40.1 yr, *SD* = 21.8, age range 18–90 yr) (Cole et al., 2018). T1-weighted MRI scans were first segmented into grey matter and white matter. These images were then normalized into a common space through nonlinear spatial registration. After normalization, the grey matter and white matter images were concatenated and transformed into a similarity matrix of the training subjects’ data, which was used to predict chronological age using a Gaussian process regression model. The model’s accuracy was evaluated through tenfold cross-validation, comparing brain-predicted age to chronological age. We applied this model to our cohort to estimate each participant’s predicted brain age using their structural imaging data (T1). To account for variability in chronological age, we computed a relative measure, which we will refer to as brain-age gap, in the following way: (predicted brain age − chronological age)/chronological age. The resulting measure—brain-age gap—is not correlated with chronological age (*r* < 0.001), allowing us to include both chronological age and brain-age gap in the same statistical models without introducing multicollinearity issues. Positive brain-age gap values indicate accelerated brain ageing while negative values reflect delayed brain ageing. This information is visualized in Figure 2.

### Language Tasks

We report information from two comprehension and two production tasks. The tasks are summarized in Figure 1 and Table 1.

#### Listening comprehension

This task explored age-related differences in the use of syntactic and semantic information during sentence comprehension. Participants engaged in a speech monitoring task, where they listened to spoken sentences and were asked to press a button as quickly as possible upon hearing a target word (e.g., spatula). The sentences varied in structure: They could be lists of words in random order, low-constraint sentences, or high-constraint sentences (see Table 1 for examples).

Shorter word monitoring response times (RTs) suggest easier lexical access driven by expectations derived from different types of linguistic representations that listeners build incrementally word by word. The difference between the random-word order and low-constraint conditions indexes the use of syntactic cues, while the contrast between low- and high-constraint conditions indexes the role of semantic information.

The stimuli for this experiment included 60 target items, each integrated within sentence contexts. The targets were presented either in a low-constraining context, a high-constraining context, or in the random-word order condition (the latter was generated by randomizing the words from the low-constraint sentences). The stimuli were pretested in a cloze task to confirm differences in predictability across conditions.

Each of the 60 items appeared in all three context conditions across three separate lists (using a Latin square design). To ensure variety, 12 additional filler items with different target words/sentences were included. Thus, each list consisted of 72 trials, divided into four blocks of 18 items (15 experimental and 3 filler items per block). Four practice sentences were presented at the beginning of the experiment, prior to the main trials. All sentences were recorded by a female native speaker of Standard British English. Each trial began with a fixation cross (+) displayed for 500 ms, followed by a 1,000 ms blank screen, after which the target word appeared on screen for 1,000 ms. Half a second after the target word ended, the spoken sentence began. Participants were asked to monitor the auditory input for the visually presented target word and to press the space bar when they detected the target. Each trial concluded 2,000 ms after the audio file ended. RTs were recorded from the onset of the target word in the spoken sentence.

#### Reading comprehension

In the reading comprehension task, participants were visually presented with sentences one at a time. Participants were instructed to press the space bar when they had finished reading the sentence, after which they responded to a comprehension question assessing the identity of the agent or patient referenced in the sentences (e.g., Who is being blessed?). The two animate nouns, “boy” and “girl” (which remained constant across all items), were displayed below the question, and participants had to choose the correct answer by pressing either “A” or “L” on the keyboard.

The sentences varied across four levels of increasing syntactic complexity, as illustrated in Table 1. A total of 24 sentences were used, organized into four lists, each containing six items from each condition. The sentences were rotated across conditions using a Latin square design so that every item appeared in all four conditions across the different lists. The initial four lists were duplicated, with the item order rearranged to create four additional lists. Participants were randomly assigned to one of the eight lists. Before the main task, participants completed four practice trials featuring sentences with similar syntactic structures to the experimental items.

#### Tip-of-the-tongue task

In this task, definitions were visually presented alongside three response options: (1) “Know,” (2) “Don’t Know,” and (3) “ToT.” Participants were asked to select Know if they knew the word the definition was referring to, Don’t Know if they did not know, and ToT if they experienced a tip-of-the-tongue state. We calculated the proportion of tip-of-the tongue responses by dividing the amount of true tip-of-the-tongues by the total number of trials. We only considered a trial as a true tip-of-the-tongue if the participants answered they experienced a tip-of-the-tongue state *and* pressed “yes” when subsequently asked, “Is this the word you were thinking of?”

Therefore, trial length varied depending on whether a tip-of-the-tongue was reported (as only on those trials an additional verification slide was presented to the participant). Each definition was shown for 12,000 ms followed by a 2,000 ms interstimulus interval. Verification slides (only following tip-of-the-tongue responses) were presented for a maximum of 6,000 ms (they disappeared as soon as the participants answered with “yes” or “no”). A jittered intertrial inverval was used with an average of 5,750 ms (range 3,500–8,000 ms; 500 ms increments). 200 unique definitions were displayed in total, split over four blocks (50 definitions per block). We counterbalanced correct target responses across those four blocks to match for the number of proper and common nouns and average syllable, phoneme, letter count and word frequency.

#### Phrase production

This task assessed utterance planning scope. Participants were asked to describe two objects within one phrase, with the objects’ location manipulated such that participants produced phrase types known to have differing planning scopes: coordinate noun phrases (e.g., “The cone and the grape”) and noun phrases (NPs) modified by prepositional phrases (e.g., “The cone above the grape”). We further manipulated complexity by having the second NP modified or not modified by an adjective (simple vs. complex; e.g., “The cone and the grape” vs. “The cone and the pink grape”). The speech onset is believed to be a measure of the amount of preparation that is needed to start producing utterances, that is, planning scope (e.g., Allum & Wheeldon, 2007; Konopka & Meyer, 2014). Longer onsets are observed for coordinate than for prepositional phrases (Allum & Wheeldon, 2007) and for more versus less complex utterance.

On each trial, four pictures were displayed. Two of the pictures were surrounded by a rectangular red line frame, indicating they were the target words to be used in the to-be-produced phrase. Coordinate phrases were cued by a horizontal red frame (which could either appear at the top or at the bottom), and prepositional phrases were cued by a vertical red frame (which could either appear on the right or on the left). The second NP was either simple or complex (i.e., modified by an adjective). A complex second NP was elicited by making this target appear twice, once as a colour-modified target and once as the original (Figure 1D). The design crossed phrase type (coordinate; prepositional) and complexity (simple: not modified; complex: adjective modified).

The materials included 20 target pictures selected from the MultiPic database (Duñabeitia et al., 2018), combined in 20 unique word-pairs to make up a phrase (each picture occurred both as the first or second target in a word-pair). Each of the 20 word-pair items appeared in the four experimental conditions such that each participant experienced every item in each condition. As such, 80 experimental items were divided across four conditions, with each individual experimental image presented 8 times (rotated across screen locations). The other two of the four pictures were images that did not appear in another experimental item but only as part of the filler displays (we had 48 filler items).

Prior to this experiment, participants completed two practice blocks (based on the 20 experimental pictures rearranged and 16 fillers). A central fixation cross (+) was displayed at the beginning of each trial for 500 ms, followed by a 500 ms blank screen, after which the multipicture display was shown and recording of phrase production started. We automatically registered speech onset, while the experimenter recorded response accuracy (i.e., responses in which participants did not use the expected names or phrase type, where they did not mention the adjective, or disfluent responses were all categorized as errors). The trial finished after the participant stopped speaking (or after 3,000 ms of silence).

### Additional Measures

#### Education

Education has been suggested as a protective factor against cognitive decline (Tucker & Stern, 2011; Zahodne et al., 2015) and as a predictor of language performance (Béland et al., 1993; Le Dorze & Bédard, 1998; Mackenzie, 2000). Consequently, we included it as a control variable in all our models. Education was treated as a binary variable, where participants with university-level education or higher were assigned a value of 1, and those without college-level education were assigned a value of 0. We also tested alternative coding approaches (e.g., using years of education as a continuous variable or education level as a categorical variable), but these did not affect the results.

#### Working memory

Given that working memory is associated with both ageing and syntactic and semantic processing (DeDe et al., 2004; Waters & Caplan, 2001), we included it as a control variable in models analyzing tasks that manipulate semantic and/or syntactic complexity, specifically listening comprehension, reading comprehension, and phrase production. We operationalized working memory based on participants’ performance on a digit span task.

In the digit span task, participants were shown single digits (0–9) one at a time on a computer screen, with each digit appearing for 1,000 ms. The sequences started with three digits and could extend up to 12 digits. After the entire sequence was displayed, participants were required to type the digits in the exact order they appeared using the computer keyboard, pressing the Enter key to confirm their entry. Feedback was provided after each trial. Each level consisted of three trials, and participants had to correctly complete two out of three trials to advance to the next level. The task automatically terminated if the participant made two incorrect responses within a level. The highest level where the participant correctly completed two trials determines their digit span. Measuring working memory using Digit Span tests is a common method in psychological research (Feier & Gerstman, 1980; Grégoire & Van der Linden, 1997). The entire task took approximately 5 min to complete.

#### Vocabulary size

Vocabulary size estimations were based on a custom vocabulary task in which participants were presented with 30 words for which they had to select either a synonym or an antonym between four options (mean = 77.22, *SD* = 11.89). This measure was used as regressor in the analysis of the tip-of-the-tongue task.

### Preprocessing and Analysis

We first refined our datasets for each task by retaining only those participants who had available T1-weighted MRI scans, as well as the required demographic and behavioural information. This resulted in the following final participant counts for each task: *N* = 85 for listening comprehension, *N* = 80 for reading comprehension, *N* = 80 for tip-of-the-tongue, and *N* = 82 for phrase production. To exclude implausibly fast or slow responses, we then removed trials with RTs below 150 ms and above 1,500 ms in the listening comprehension task and below 250 ms and above 2,500 ms in the phrase production task (see Fernandes, Segaert, et al., 2025, for the justification of the trimming procedure, where the same tasks were used). RTs were then log-transformed to reduce positive skew in the data. We also removed RTs which were more than 2.5 standard deviations from the mean in the listening comprehension, reading comprehension, and phrase production tasks. Additionally, in the phrase production task we removed incorrect trials (e.g., wrong syntax, missing/wrong adjective, incorrect noun, hesitation; 23.4%). These preprocessing steps resulted in the removal of 4.9% of data in the listening comprehension task, 1.72% of data in the reading comprehension task and 28.3% of data in the phrase production task.

We analyzed our data using Bayesian mixed-effects models (as implemented in the brms package in R; Bürkner, 2017). We summarized the evidence for a parameter estimate along with its 95% probability intervals (also known as credible intervals). We calculated Bayes factors (BFs; Wagenmakers et al., 2018) to assess the strength of the evidence in favor of the alternative hypothesis over the null hypothesis. For example, a BF of 1 means that evidence for either hypothesis is equally strong, values above 3 provide moderate evidence and values above 10 show strong evidence (e.g., Dickey & Lientz, 1970; Wagenmakers et al., 2010). In addition, BFs also allow us to test the evidence in favor of the null hypothesis which is not possible with traditional inferential methods (Dienes, 2014; Schad et al., 2023).

Model specifications are summarized in Table 3. All models included brain-age gap, chronological age, and education as fixed effects. Additionally, for listening comprehension, reading comprehension, and phrase production, a measure of working memory (performance on a digit span task) was included. For the tip-of-the-tongue task, target word frequencies and length as well as participants’ vocabulary size were included. Random intercepts were included for participants and items. When required by the design, random slopes for linguistic manipulations were also included for participants and items. Continuous predictors (brain-age gap, chronological age, digit span, frequency, length, and vocabulary size) were standardized. For the listening and reading comprehension tasks, categorical predictors were sum coded.

**Table 3.** Model specifications for the four experimental tasks

**Task**	**Model specification**
**Listening comprehension**	log(RT) ∼ Semantics + Syntax + Digit span + Education + Brain-age gap + Age + Brain-age gap : (Semantic + Syntax) + Age : (Semantics + Syntax) + (Semantics + Syntax | Participant) + (Semantics + Syntax | Item)
**Reading comprehension**	logit(Accuracy) ∼ Complexity + Digit Span + Education + Brain-age gap + Age + Brain-age gap : Complexity + Age : Complexity + (Complexity | Participant) + (Complexity | Item)
**Tip-of-the-tongue**	logit(ToT) ∼ Length + Frequency + Vocabulary size + Education + Brain-age gap + Age + (1 | Participant) + (1 | Item)
**Phrase production**	log(RT) ∼ Phrase type + Complexity + Digit span + Education + Brain-age gap + Age + Brain-age gap : Phrase type + Brain-age gap : Complexity + Age : Phrase type + Age : Complexity + Phrase type : Complexity + (Phrase type + Complexity | Participant) + (Phrase type + Complexity | Item)

*Note*. Model specifications presented using expressions similar to the formula syntax of brms in R. For respons times (RT) measures we used log-Gaussian distributions. For reading comprehension accuracy and tip-of-the-tongue (ToT) data we used Bernoulli distributions. Colons indicate interactions.

## RESULTS

### Listening Comprehension

**Figure 3.**
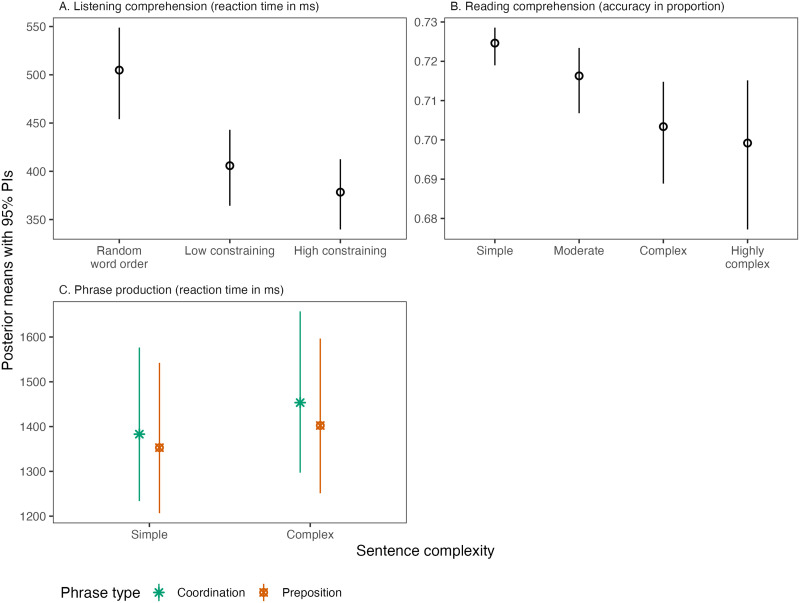
Modeled posterior estimates. (A) Reaction times (in ms) in the listening comprehension task. (B) Response accuracy (in proportions) in the reading comprehension task. (C) Reaction times (in ms) in the phrase production task. Error bars represent 95% probability intervals (PIs).

Listening comprehension was substantially faster for sentences with syntactic structure and constraining semantic meaning (BF10s > 100, see Figure 3A). No evidence was found for effects of digit span, education, or brain-age gap. Linguistic structure of the stimulus did not interact with age and brain-age gap. (Evidence for the null hypothesis of all interactions was strong; BF01s > 10.) All model coefficients can be found in Table 4.

**Table 4.** Bayes factors and estimates of predictor coefficients in the listening comprehension task

**Predictor**	**Estimate**	**PI**	**BF01**	**BF10**
Semantics: high vs. low-constraining	−0.07	−0.1–−0.04	<0.01	>100
Syntax: low-constraining vs. random word order	−0.22	−0.25–−0.19	<0.01	>100
Age	0.02	−0.02–0.06	27.94	0.04
Brain-age gap	−0.02	−0.06–0.02	25.3	0.04
Digit span	−0.01	−0.05–0.02	41.4	0.02
Education	0.04	−0.03–0.11	15.78	0.06
Age: Semantics	0.00	−0.01–0.02	>100	0.01
Age: Syntax	−0.00	−0.03–0.02	90.9	0.01
Brain-age gap: Semantics	0.01	0–0.03	28.41	0.04
Brain-age gap: Syntax	−0.02	−0.04–0.01	32.39	0.03

*Note*. The table shows the posterior estimates, 95% probability intervals, and Bayes factors (BF01 and BF10) for each predictor. BF01 represents the evidence in favor of the null hypothesis and BF10 represents evidence favoring the alternative hypothesis. As a rule of thumb, values larger than 3 indicate support for either hypothesis.

### Reading Comprehension

The analysis revealed evidence for longer reading times for increased sentence complexity (simple vs. moderate: BF10 = 6.68; moderate vs. complex: BF10 = 5.41) but with no further increase for highly complex phrases (BF10 = 0.35; see Figure 3B). Performance on the digit span task predicted accuracy on the reading comprehension task, with higher digit span scores being positively associated with greater reading comprehension accuracy (BF10 = 11.43). Effects of education, brain-age gap, age, and their interactions with sentence complexity were negligible. Model coefficients are summarized in Table 5.

**Table 5.** Bayes factors and estimates of predictor coefficients in the reading comprehension task

**Predictor**	**Estimate**	**PI**	**BF01**	**BF10**
Complexity 1: simple vs. moderate	−0.90	−1.73–−0.17	0.15	6.68
Complexity 2: moderate vs. complex	−0.70	−1.3–−0.13	0.18	5.41
Complexity 3: complex vs. highly complex	0.14	−0.52–0.81	2.82	0.35
Brain-age gap	0.26	−0.06–0.59	1.56	0.64
Age	0.17	−0.2–0.53	3.79	0.26
Education	0.46	−0.11–1.04	1.03	0.97
Digit span	0.49	0.18–0.82	0.09	11.43
Brain-age gap: Complexity 1	0.43	−0.12–1	1.10	0.91
Brain-age gap: Complexity 2	−0.36	−0.83–0.12	1.31	0.76
Brain-age gap: Complexity 3	−0.13	−0.6–0.3	3.53	0.28
Age: Complexity 1	−0.09	−0.75–0.6	2.94	0.34
Age: Complexity 2	0.00	−0.54–0.53	3.65	0.27
Age: Complexity 3	−0.16	−0.68–0.35	3.15	0.32

*Note*. The table shows the posterior estimates, 95% probability intervals, and Bayes factors (BF01 and BF10) for each predictor. BF01 represents the evidence in favor of the null hypothesis and BF10 represents evidence favoring the alternative hypothesis. Values larger than 3 indicate evidence in support of either hypothesis.

### Tip-of-the-Tongue

A mean of 59% (*SD* = 13%) of responses were recorded as known, and 17% (*SD* = 7%) were in the tip-of-the tongue state. Unknown words were removed from the analysis (mean = 22%, *SD* = 10%). There was weak evidence for a negative effect of frequency (BF10 = 2.31). For all other predictors we found moderate (BF01s > 4) to strong (BF01 > 10) evidence for the null hypothesis. A summary of all predictor coefficients can be found in Table 6.

**Table 6.** Bayes factors and estimates of predictor coefficients in the tip-of-the-tongue task

**Predictor**	**Estimate**	**PI**	**BF01**	**BF10**
Brain-age gap	0.07	−0.1–0.23	9.37	0.11
Education	−0.08	−0.46–0.29	5.39	0.19
Age	0.12	−0.06–0.31	4.68	0.21
Frequency	−0.15	−0.25–−0.04	0.43	2.31
Length (# of phonemes)	0.05	−0.05–0.15	14.21	0.07
Vocabulary size	−0.10	−0.29–0.09	6.02	0.17

*Note*. The table shows the posterior estimates, 95% probability intervals, and Bayes factors (BF01 and BF10) for each predictor. BF01 represents the evidence in favor of the null hypothesis and BF10 represents evidence favoring the alternative hypothesis. Values larger than 3 indicate evidence in support of either hypothesis.

### Phrase Production

The results (summarized in Table 7 and Figure 3C) revealed evidence for phrase type (BF10 = 7.9) and complexity (BF10 > 100), whereby coordinate phrases took longer to produce than prepositional phrases, and complex phrases (phrases with adjectives) took longer to produce than simple phrases. For all other predictors we found moderate (BF01s > 8) to strong (BF01 > 10) evidence for the null hypothesis.

**Table 7.** Bayes factors and estimates of predictor coefficients in the phrase production task

**Predictor**	**Estimate**	**PI**	**BF01**	**BF10**
Phrase type	−0.04	−0.06–−0.02	0.13	7.9
Complexity	−0.05	−0.07–−0.03	0	>100
Brain-age gap	−0.02	−0.07–0.03	28.37	0.04
Education	0.06	−0.03–0.15	8.57	0.12
Digit span	0.02	−0.02–0.07	28.25	0.04
Age	0.02	−0.02–0.07	27.8	0.04
Brain-age gap: Phrase type	0.00	−0.01–0.02	>100	0.01
Brain-age gap: Complexity	−0.01	−0.02–0	40.63	0.02
Age: Phrase type	−0.00	−0.01–0.01	>100	0.01
Age: Complexity	0.00	−0.01–0.01	>100	0.01
Phrase type: Complexity	0.01	−0.01–0.03	40.22	0.02

*Note*. The table shows the posterior estimates, 95% probability intervals, and Bayes factors (BF01 and BF10) for each predictor. BF01 represents the evidence in favor of the null hypothesis and BF10 represents evidence favoring the alternative hypothesis. Values larger than 3 indicate evidence in support of either hypothesis.

## DISCUSSION

We tested whether the extent to which the modeled age of a participant’s brain deviates from chronological age (i.e., brain-age gap) explains individual differences in language abilities in healthy older adults. Brain-age related effects have previously been linked to individual differences in various cognitive functions (Cole et al., 2018; Elliott et al., 2021) in both healthy and pathological ageing, and to language abilities in pathological ageing (Kristinsson et al., 2022). We used Bayesian inferential methods to analyze four established measures of language comprehension and production abilities of a relatively large sample of healthy older participants (*n* = 86; 60 to 81 years). Our results provide consistent evidence that brain-age gap does not predict language performance in healthy older adults, which challenges the idea that brain age is a reliable determinant of language processing.

Effects of the linguistic manipulations within our comprehensive set of language tasks were as predicted: In the listening comprehension task, the presence of syntactic structure facilitated target word recognition (compared to the random word order condition) and participants further benefited from having a more predictive semantic context. In the reading comprehension task, accuracy declined as syntactic complexity increased. Additionally, higher working memory capacity was associated with better overall comprehension accuracy. In the picture description task, prepositional phrases (as opposed to coordinate phrases) and simpler syntactic structures led to faster naming onset times. And finally, in the tip-of-the-tongue task, the proportion of observed tip-of-the-tongue responses was consistent with what is typically expected in such tasks. Each of the tasks thus showed effects which were predicted and in line with previous literature (Burke & Shafto, 2008; Peelle, 2019).

Our results are unlikely to be a failure to find an effect because of methodological limitations. This is for three reasons: First, our study has a relatively large sample size (for comparison, Kristinsson et al., 2022, found robust effects with 49 participants). Second, Bayesian tools allow for robust conclusions about the absence of an effect (Dienes, 2014; Wagenmakers et al., 2018). Third, we used a comprehensive set of language tasks that have previously demonstrated sensitivity to ageing and individual variability in language abilities between older adults (Caplan et al., 2011; Caplan & Waters, 2005; Fernandes, Segaert, et al., 2025; Hardy et al., 2020; Segaert et al., 2018; Waters & Caplan, 2001). Yet, despite robust task effects, there was strong evidence against relationships with brain-age gap.

Given the mixed findings in the literature, it is perhaps not surprising that our study did not detect an effect of chronological age on language processing performance (Fernandes, Segaert, et al., 2025; Tyler et al., 2010). Such variability across studies may be attributed to differences in age ranges, processing contexts, task-specific demands, and potential methodological limitations. For example, some changes in language processing may be more strongly associated with neurodegenerative or otherwise atypical ageing decline, rather than healthy ageing. Our sample, however, included only cognitively healthy individuals. Another possible explanation for the absence of chronological age effects in our study is the limited age range of the participants (60–81 yr old, with 86% 60–70 yr old). Chronological age effects may be more pronounced when considering age as a continuous variable across a broader age range (Brysbaert, 2024), or, when comparing younger to older adults. For example, Fernandes, Segaert, et al. (2025) found age-related effects in the same phrase production task data when comparing older adults to younger adults, whereby older speakers were slower than younger speakers in producing small-scope prepositional phrases (e.g., “the cone above the grape”), which suggests that older adults may engage in more extensive planning. They also reported that older adults generally outperformed younger adults on high-constraint sentences in the listening comprehension task, arguably because they have accumulated word and world knowledge. While our study did not include a younger comparison group (since there was no structural brain data available for them), we made use of the available data to examine individual differences within the age range that was accessible to us.

Effects of brain-age gap on language performance are, to some extent, independent of chronological age effects. In other words, the absence of effects of chronological age on language performance does not have to coincide with the absence of brain-age gap effects. While chronological age is commonly used as proxy for cognitive ageing, brain-age gap was introduced in the literature to provide a more individualized measure of brain health that captures more nuanced aspects of the ageing process beyond age alone. Maintenance models of healthy ageing put forth that neural resources can be maintained or restored to their former levels in response to the typical “wear and tear” associated with nonpathological ageing (Habeck et al., 2017; Nyberg et al., 2012). Factors such as genetics, environment, and lifestyle can promote such maintenance, supporting brain and cognitive functions (Cabeza et al., 2018), explaining why there is wide interindividual variability within the healthy older adult population. Much of the literature linking brain age to cognitive outcomes has focused on clinical groups, such as individuals with mild cognitive impairment or Alzheimer’s disease, where the effects of brain ageing are more pronounced. In contrast, healthy older adults may have greater potential for mechanisms of maintenance which may weaken the association between brain age estimates and specific linguistic outcomes in healthy older individuals. Using a multimodal approach (e.g., combining structural MRI, functional MRI, diffusion tensor imaging, or arterial spin labeling) to predict brain age may provide a more comprehensive estimate of overall brain health in healthy older adults and thus have stronger associations with cognitive performance measures (Dijsselhof et al., 2023; Liem et al., 2017; Mooraj et al., 2025).

One limitation of the present study concerns the generalizability of our findings. Our sample was relatively homogeneous, predominantly consisting of cognitively healthy, white older adults, which may limit applicability to more diverse populations. Additionally, the age range was restricted (60–81 yr, with most participants 60–70 yr), which may have affected the ability to detect age-related effects on language performance. It is possible that a wider age range may have revealed significant age-related effects on language performance.

In the present article we present consistent evidence that brain-age gap does not predict language processing, at least in healthy (as opposed to pathological) ageing, highlighting the need to consider other neural and cognitive factors when studying language decline. Individual variability in language decline within the older population is large, and the complex interactions between factors determining individuals’ ageing trajectories are difficult to quantify, which motivated our endeavor to assess the utility of a biological brain age marker as a tool with which to begin to explain this variability. Future research could aim to further explore how we can explain individual variability in older adults’ language decline using different and more sensitive approaches. These could include longitudinal designs that track changes in brain structure and language performance over time and a wider age range, or neuroimaging techniques using a multimodal approach to predict brain age. The long-term goal is to advance our understanding of cognitive ageing, including in the domain of language, ultimately contributing to targeted interventions aimed at preserving language abilities in later life.

## ACKNOWLEDGMENTS

We thank Consuelo Vidal Gran, Nicolas Hayston, Rupali Limachya, Amelie Grandjean, Aoife Marley, Shi Miao, and Samuel Thomas for data collection support.

## FUNDING INFORMATION

Yanina Prystauka, Foyzul Rahman, Jack Feron, Samuel J. E. Lucas, Allison Wetterlin, Eunice G. Fernandes, Linda Wheeldon, and Katrien Segaert, Norges Forskningsråd (https://dx.doi.org/10.13039/501100005416), Award ID: FRIPRO 300030.

## AUTHOR CONTRIBUTIONS

**Yanina Prystauka**: Conceptualization; Formal analysis; Investigation; Methodology; Visualization; Writing – original draft; Writing – review & editing. **Foyzul Rahman**: Conceptualization; Investigation; Methodology; Writing – review & editing. **Natalie Busby**: Conceptualization; Methodology. **Jens Roeser**: Formal analysis; Methodology; Visualization; Writing – review & editing. **Carl-Johan Boraxbekk**: Conceptualization; Writing – review & editing. **Jack Feron**: Investigation; Methodology; Writing – review & editing. **Samuel J. E. Lucas**: Methodology; Writing – review & editing. **Allison Wetterlin**: Funding acquisition; Writing – review & editing. **Eunice G. Fernandes**: Investigation; Writing – review & editing. **Linda Wheeldon**: Conceptualization; Funding acquisition; Investigation; Writing – review & editing. **Katrien Segaert**: Conceptualization; Funding acquisition; Investigation; Writing – original draft; Writing – review & editing.

## DATA AND CODE AVAILABILITY STATEMENT

The data for the present study were collected as part of a larger study (preregistration: https://osf.io/6fqg7. Materials and data for the present report are at https://github.com/yanina-prystauka/FAB_BrainAge).

## TECHNICAL TERMS

Brain-age gap: The difference between a person’s chronological age and their predicted brain age, estimated using neuroimaging data and machine learning.
Tip-of-the-tongue: A cognitive phenomenon in which someone is experiencing difficulty in producing a known or intended word, often retrieving partial information.
Bayes factors (BFs): A statistical tool used in Bayesian inference to compare the strength of evidence for two competing hypotheses.

## References

[bib1] Allum, P. H., & Wheeldon, L. R. (2007). Planning scope in spoken sentence production: The role of grammatical units. Journal of Experimental Psychology: Learning, Memory, and Cognition, 33(4), 791–810. 10.1037/0278-7393.33.4.791, 17576154

[bib2] Baril, A.-A., Beiser, A. S., Mysliwiec, V., Sanchez, E., DeCarli, C. S., Redline, S., Gottlieb, D. J., Mailard, P., Romero, J. R., Satizabal, C. L., Zucker, J. M., Seshadri, S., Pase, M. P., & Himali, J. J. (2021). Slow-wave sleep and MRI markers of brain aging in a community-based sample. Neurology, 96(10), e1462–e1469. 10.1212/WNL.0000000000011377, 33361258 PMC8055313

[bib3] Béland, R., Lecours, A. R., Giroux, F., & Bois, M. (1993). The MT-86 *β* aphasia battery: A subset of normative data in relation to age and level of school education (part II). Aphasiology, 7(4), 359–382. 10.1080/02687039308249516

[bib4] Brysbaert, M. (2024). Designing and evaluating tasks to measure individual differences in experimental psychology: A tutorial. Cognitive Research: Principles and Implications, 9(1), Article 11. 10.1186/s41235-024-00540-2, 38411837 PMC10899130

[bib6] Burke, D. M., Locantore, J. K., Austin, A. A., & Chae, B. (2004). Cherry pit primes Brad Pitt: Homophone priming effects on young and older adults’ production of proper names. Psychological Science, 15(3), 164–170. 10.1111/j.0956-7976.2004.01503004.x, 15016287 PMC2255560

[bib7] Burke, D. M., MacKay, D. G., Worthley, J. S., & Wade, E. (1991). On the tip of the tongue: What causes word finding failures in young and older adults? Journal of Memory and Language, 30(5), 542–579. 10.1016/0749-596X(91)90026-G

[bib5] Burke, D. M., & Shafto, M. A. (2008). Language and aging. In F. I. M. Craik & T. A. Salthouse (Eds.), The handbook of aging and cognition (3rd ed., pp. 373–443). Psychology Press.

[bib8] Bürkner, P.-C. (2017). brms: An R package for Bayesian multilevel models using Stan. Journal of Statistical Software, 80(1), 1–28. 10.18637/jss.v080.i01

[bib9] Busby, N., Newman-Norlund, S., Sayers, S., Newman-Norlund, R., Wilmskoetter, J., Rorden, C., Nemati, S., Wilson, S., Riccardi, N., Roth, R., Johnson, L., den Ouden, D. B., Fridriksson, J., & Bonilha, L. (2023). Lower socioeconomic status is associated with premature brain aging. Neurobiology of Aging, 130, 135–140. 10.1016/j.neurobiolaging.2023.06.012, 37506551 PMC13277732

[bib10] Cabeza, R., Albert, M., Belleville, S., Craik, F. I. M., Duarte, A., Grady, C. L., Lindenberger, U., Nyberg, L., Part, D. C., Reuter-Lorenz, P. A., Rugg, M. D., Steffener, J., & Rajah, M. N. (2018). Maintenance, reserve and compensation: The cognitive neuroscience of healthy ageing. Nature Reviews Neuroscience, 19(11), 701–710. 10.1038/s41583-018-0068-2, 30305711 PMC6472256

[bib11] Caplan, D., DeDe, G., Waters, G., Michaud, J., & Tripodis, Y. (2011). Effects of age, speed of processing, and working memory on comprehension of sentences with relative clauses. Psychology and Aging, 26(2), 439–450. 10.1037/a0021837, 21480714

[bib12] Caplan, D., & Waters, G. (2005). The relationship between age, processing speed, working memory capacity, and language comprehension. Memory, 13(3–4), 403–413. 10.1080/09658210344000459, 15952262

[bib13] Carson, N., Leach, L., & Murphy, K. J. (2018). A re-examination of Montreal Cognitive Assessment (MoCA) cutoff scores. International Journal of Geriatric Psychiatry, 33(2), 379–388. 10.1002/gps.4756, 28731508

[bib14] Charlton, R. A., Barrick, T. R., McIntyre, D. J., Shen, Y., O’Sullivan, M., Howe, F. A., Clark, C. A., Morris, R. G., & Markus, H. S. (2006). White matter damage on diffusion tensor imaging correlates with age-related cognitive decline. Neurology, 66(2), 217–222. 10.1212/01.wnl.0000194256.15247.83, 16434657

[bib15] Cole, J. H. (2020). Multimodality neuroimaging brain-age in UK biobank: Relationship to biomedical, lifestyle, and cognitive factors. Neurobiology of Aging, 92, 34–42. 10.1016/j.neurobiolaging.2020.03.014, 32380363 PMC7280786

[bib16] Cole, J. H., & Franke, K. (2017). Predicting age using neuroimaging: Innovative brain ageing biomarkers. Trends in Neurosciences, 40(12), 681–690. 10.1016/j.tins.2017.10.001, 29074032

[bib17] Cole, J. H., Ritchie, S. J., Bastin, M. E., Valdés Hernández, M. C., Muñoz Maniega, S., Royle, N., Corley, J., Pattie, A., Harris, S. E., Zhang, Q., Wray, N. R., Redmond, P., Marioni, R. E., Starr, J. M., Cox, S. R., Wardlaw, J. M., Sharp, D. J., & Deary, I. J. (2018). Brain age predicts mortality. Molecular Psychiatry, 23(5), 1385–1392. 10.1038/mp.2017.62, 28439103 PMC5984097

[bib18] DeDe, G., Caplan, D., Kemtes, K., & Waters, G. (2004). The relationship between age, verbal working memory, and language comprehension. Psychology and Aging, 19(4), 601–616. 10.1037/0882-7974.19.4.601, 15584786

[bib19] Diaz, M. T., Rizio, A. A., & Zhuang, J. (2016). The neural language systems that support healthy aging: Integrating function, structure, and behavior. Language and Linguistics Compass, 10(7), 314–334. 10.1111/lnc3.12199, 28210287 PMC5304920

[bib20] Dickey, J. M., & Lientz, B. P. (1970). The weighted likelihood ratio, sharp hypotheses about chances, the order of a Markov chain. Annals of Mathematical Statistics, 41(1), 214–226. 10.1214/aoms/1177697203

[bib21] Dienes, Z. (2014). Using Bayes to get the most out of non-significant results. Frontiers in Psychology, 5, Article 781. 10.3389/fpsyg.2014.00781, 25120503 PMC4114196

[bib22] Dijsselhof, M. B. J., Barboure, M., Stritt, M., Nordhøy, W., Wink, A. M., Beck, D., Westlye, L. T., Cole, J. H., Barkhof, F., Mustsaert, H. J. M. M., & Petr, J. (2023). The value of arterial spin labelling perfusion MRI in brain age prediction. Human Brain Mapping, 44(7), 2754–2766. 10.1002/hbm.26242, 36852443 PMC10089088

[bib23] Duñabeitia, J. A., Crepaldi, D., Meyer, A. S., New, B., Pliatsikas, C., Smolka, E., & Brysbaert, M. (2018). MultiPic: A standardized set of 750 drawings with norms for six European languages. Quarterly Journal of Experimental Psychology, 71(4), 808–816. 10.1080/17470218.2017.1310261, 28326995

[bib24] Dunås, T., Wåhlin, A., Nyberg, L., & Boraxbekk, C.-J. (2021). Multimodal image analysis of apparent brain age identifies physical fitness as predictor of brain maintenance. Cerebral Cortex, 31(7), 3393–3407. 10.1093/cercor/bhab019, 33690853 PMC8196254

[bib25] Elliott, M. L., Belsky, D. W., Knodt, A. R., Ireland, D., Melzer, T. R., Poulton, R., Ramrakha, S., Caspi, A., Moffitt, T. E., & Hariri, A. R. (2021). Brain-age in midlife is associated with accelerated biological aging and cognitive decline in a longitudinal birth cohort. Molecular Psychiatry, 26(8), 3829–3838. 10.1038/s41380-019-0626-7, 31822815 PMC7282987

[bib26] Federmeier, K. D., & Kutas, M. (2005). Aging in context: Age-related changes in context use during language comprehension. Psychophysiology, 42(2), 133–141. 10.1111/j.1469-8986.2005.00274.x, 15787850

[bib27] Federmeier, K. D., Kutas, M., & Schul, R. (2010). Age-related and individual differences in the use of prediction during language comprehension. Brain and Language, 115(3), 149–161. 10.1016/j.bandl.2010.07.006, 20728207 PMC2975864

[bib28] Federmeier, K. D., McLennan, D. B., de Ochoa, E., & Kutas, M. (2002). The impact of semantic memory organization and sentence context information on spoken language processing by younger and older adults: An ERP study. Psychophysiology, 39(2), 133–146. 10.1111/1469-8986.3920133, 12212662

[bib29] Federmeier, K. D., Van Petten, C., Schwartz, T. J., & Kutas, M. (2003). Sounds, words, sentences: Age-related changes across levels of language processing. Psychology and Aging, 18(4), 858–872. 10.1037/0882-7974.18.4.858, 14692871

[bib30] Feier, C. D., & Gerstman, L. J. (1980). Sentence comprehension abilities throughout the adult life span. Journal of Gerontology, 35(5), 722–728. 10.1093/geronj/35.5.722, 7430569

[bib31] Fernandes, E. G., Fosstveit, S. H., Feron, J., Rahman, F., Lucas, S. J. E., Lohne-Seiler, H., Berntsen, S., Wetterlin, A., Segaert, K., & Wheeldon, L. (2025). Effects of increasing fitness through exercise training on language comprehension in monolingual and bilingual older adults: A randomized controlled trial. Aging, Neuropsychology, and Cognition, 32(4), 485–517. 10.1080/13825585.2024.2435914, 39693229

[bib32] Fernandes, E. G., Segaert, K., Rahman, F., Wetterlin, A., & Wheeldon, L. (2025). Bilingualism and ageing independently impact on language processing: Evidence from comprehension and production. Bilingualism: Language and Cognition, 28(3), 778–792. 10.1017/S1366728924000245

[bib33] Feron, J., Rahman, F., Fosstveit, S. H., Joyce, K. E., Gilani, A., Lohne-Seiler, H., Berntsen, S., Mullinger, K. J., Segaert, K., & Lucas, S. J. E. (2024). Cerebral blood flow and arterial transit time responses to exercise training in older adults. NeuroImage, 303, Article 120919. 10.1016/j.neuroimage.2024.120919, 39505224

[bib34] Feron, J., Segaert, K., Rahman, R., Fosstveit, S. H., Joyce, K. E., Gilani, A., Lohne-Seiler, H., Berntsen, S., Mullinger, K. J., & Lucas, S. J. E. (2024). Determinants of cerebral blood flow and arterial transit time in healthy older adults. Aging, 16(18), 12473–12497. 10.18632/aging.206112, 39302230 PMC11466485

[bib35] Ferreira, D., Molina, Y., Machado, A., Westman, E., Wahlund, L.-O., Nieto, A., Correia, R., Junqué, C., Díaz-Flores, L., & Barroso, J. (2014). Cognitive decline is mediated by gray matter changes during middle age. Neurobiology of Aging, 35(5), 1086–1094. 10.1016/j.neurobiolaging.2013.10.095, 24239436

[bib36] Ferrucci, L., Gonzalez-Freire, M., Fabbri, E., Simonsick, E., Tanaka, T., Moore, Z., Salimi, S., Sierra, F., & de Cabo, R. (2020). Measuring biological aging in humans: A quest. Aging Cell, 19(2), Article e13080. 10.1111/acel.13080, 31833194 PMC6996955

[bib37] Fjell, A. M., & Walhovd, K. B. (2010). Structural brain changes in aging: Courses, causes and cognitive consequences. Reviews in the Neurosciences, 21(3), 187–221. 10.1515/REVNEURO.2010.21.3.187, 20879692

[bib38] Folstein, M. F., Folstein, S. E., & McHugh, P. R. (1975). “Mini-mental state”: A practical method for grading the cognitive state of patients for the clinician. Journal of Psychiatric Research, 12(3), 189–198. 10.1016/0022-3956(75)90026-6, 1202204

[bib39] Fosstveit, S. H., Berntsen, S., Feron, J., Joyce, K. E., Ivarsson, A., Segaert, K., Lucas, S. J. E., & Lohne-Seiler, H. (2024). HIIT at home: Enhancing cardiorespiratory fitness in older adults—A randomised controlled trial. Scandinavian Journal of Medicine & Science in Sports, 34(7), Article e14694. 10.1111/sms.14694, 38982665

[bib40] Franke, K., & Gaser, C. (2012). Longitudinal changes in individual *BrainAGE* in healthy aging, mild cognitive impairment, and Alzheimer’s disease. GeroPsych, 25(4), 235–245. 10.1024/1662-9647/a000074

[bib41] Fratiglioni, L., Marseglia, A., & Dekhtyar, S. (2020). Ageing without dementia: Can stimulating psychosocial and lifestyle experiences make a difference? Lancet Neurology, 19(6), 533–543. 10.1016/S1474-4422(20)30039-9, 32470425

[bib42] Gollan, T. H., & Brown, A. S. (2006). From tip-of-the-tongue (TOT) data to theoretical implications in two steps: When more TOTs means better retrieval. Journal of Experimental Psychology: General, 135(3), 462–483. 10.1037/0096-3445.135.3.462, 16846276

[bib43] Grégoire, J., & Van der Linden, M. (1997). Effect of age on forward and backward digit spans. Aging, Neuropsychology, and Cognition, 4(2), 140–149. 10.1080/13825589708256642

[bib44] Habeck, C., Razlighi, Q., Gazes, Y., Barulli, D., Steffener, J., & Stern, Y. (2017). Cognitive reserve and brain maintenance: Orthogonal concepts in theory and practice. Cerebral Cortex, 27(8), 3962–3969. 10.1093/cercor/bhw208, 27405332 PMC6248534

[bib45] Hardy, S. M., Segaert, K., & Wheeldon, L. R. (2020). Healthy aging and sentence production: Disrupted lexical access in the context of intact syntactic planning. Frontiers in Psychology, 11, Article 257. 10.3389/fpsyg.2020.00257, 32153469 PMC7046760

[bib46] Hedman, A. M., van Haren, N. E. M., Schnack, H. G., Kahn, R. S., & Hulshoff Pol, H. E. (2012). Human brain changes across the life span: A review of 56 longitudinal magnetic resonance imaging studies. Human Brain Mapping, 33(8), 1987–2002. 10.1002/hbm.21334, 21915942 PMC6870052

[bib47] Heine, M. K., Ober, B. A., & Shenaut, G. K. (1999). Naturally occurring and experimentally induced tip-of-the-tongue experiences in three adult age groups. Psychology and Aging, 14(3), 445–457. 10.1037/0882-7974.14.3.445, 10509699

[bib48] Ho, A. J., Raji, C. A., Becker, J. T., Lopez, O. L., Kuller, L. H., Hua, X., Divov, I. D., Stein, J. L., Rosano, C., Toga, A. W., & Thompson, P. M. (2011). The effects of physical activity, education, and body mass index on the aging brain. Human Brain Mapping, 32(9), 1371–1382. 10.1002/hbm.21113, 20715081 PMC3184838

[bib49] Houston, J., Allendorfer, J., Nenert, R., Goodman, A. M., & Szaflarski, J. P. (2019). White matter language pathways and language performance in healthy adults across ages. Frontiers in Neuroscience, 13, Article 1185. 10.3389/fnins.2019.01185, 31736704 PMC6838008

[bib50] Kemper, S., Greiner, L. H., Marquis, J. G., Prenovost, K., & Mitzner, T. L. (2001). Language decline across the life span: Findings from the nun study. Psychology and Aging, 16(2), 227–239. 10.1037/0882-7974.16.2.227, 11405311

[bib51] Kemper, S., Herman, R., & Lian, C. (2003). Age differences in sentence production. Journals of Gerontology, Series B: Psychological Sciences and Social Sciences, 58(5), P260–P268. 10.1093/geronb/58.5.P260, 14507932

[bib52] Kemper, S., & Sumner, A. (2001). The structure of verbal abilities in young and older adults. Psychology and Aging, 16(2), 312–322. 10.1037/0882-7974.16.2.312, 11405318

[bib53] Kemper, S., Thompson, M., & Marquis, J. (2001). Longitudinal change in language production: Effects of aging and dementia on grammatical complexity and propositional content. Psychology and Aging, 16(4), 600–614. 10.1037/0882-7974.16.4.600, 11766915

[bib54] Kidd, E., Donnelly, S., & Christiansen, M. H. (2018). Individual differences in language acquisition and processing. Trends in Cognitive Sciences, 22(2), 154–169. 10.1016/j.tics.2017.11.006, 29277256

[bib55] Koini, M., Duering, M., Gesierich, B. G., Rombouts, S. A. R. B., Ropele, S., Wagner, F., Enzinger, C., & Schmidt, R. (2018). Grey-matter network disintegration as predictor of cognitive and motor function with aging. Brain Structure and Function, 223(5), 2475–2487. 10.1007/s00429-018-1642-0, 29511859 PMC5968058

[bib56] Konopka, A. E., & Meyer, A. S. (2014). Priming sentence planning. Cognitive Psychology, 73, 1–40. 10.1016/j.cogpsych.2014.04.001, 24838190

[bib57] Kristinsson, S., Busby, N., Rorden, C., Newman-Norlund, R., den Ouden, D. B., Magnusdottir, S., Hjaltason, H., Thors, H., Hillis, A. E., Kjartansson, O., Bonilha, L., & Fridriksson, J. (2022). Brain age predicts long-term recovery in post-stroke aphasia. Brain Communications, 4(5), Article fcac252. 10.1093/braincomms/fcac252, 36267328 PMC9576153

[bib58] Le Dorze, G., & Bédard, C. (1998). Effects of age and education on the lexico-semantic content of connected speech in adults. Journal of Communication Disorders, 31(1), 53–71. 10.1016/S0021-9924(97)00051-8, 9421767

[bib59] Liem, F., Varoquaux, G., Kynast, J., Beyer, F., Masouleh, S. K., Huntenburg, J. M., Lampe, L., Rahim, M., Abraham, A., Craddock, R. C., Riedel-Heller, S., Luck, T., Loeffler, M., Schroeter, M. L., Witte, A. V., Villringer, A., & Margulies, D. S. (2017). Predicting brain-age from multimodal imaging data captures cognitive impairment. NeuroImage, 148, 179–188. 10.1016/j.neuroimage.2016.11.005, 27890805

[bib60] Livingston, G., Huntley, J., Liu, K. Y., Costafreda, S. G., Selbæk, G., Alladi, S., Ames, D., Banerjee, S., Burns, A., Brayne, C., Fox, N. C., Ferri, C. P., Gitlin, L. N., Howard, R., Kales, H. C., Kivimäki, M., Larson, E. B., Nakusujja, N., Rockwood, K., … Mukadam, N. (2024). Dementia prevention, intervention, and care: 2024 report of the *Lancet* standing Commission. Lancet, 404(10452), 572–628. 10.1016/S0140-6736(24)01296-0, 39096926

[bib61] Livingston, G., Huntley, J., Sommerlad, A., Ames, D., Ballard, C., Banerjee, S., Brayne, C., Burns, A., Cohn-Mansfield, J., Cooper, C., Costafreda, S. G., Dias, A., Fox, N., Gitlin, L. N., Howard, R., Kales, H. C., Kivimäki, M., Larson, E. B., Ogunniyi, A., … Mukadam, N. (2020). Dementia prevention, intervention, and care: 2020 report of the *Lancet* Commission. Lancet, 396(10248), 413–446. 10.1016/S0140-6736(20)30367-6, 32738937 PMC7392084

[bib62] Lockhart, S. N., Mayda, A. B. V., Roach, A. E., Fletcher, E., Carmichael, O., Maillard, P., Schwarz, C. G., Yonelinas, A. P., Ranganath, C., & DeCarli, C. (2012). Episodic memory function is associated with multiple measures of white matter integrity in cognitive aging. Frontiers in Human Neuroscience, 6, Article 56. 10.3389/fnhum.2012.00056, 22438841 PMC3305887

[bib63] López-Otín, C., Blasco, M. A., Partridge, L., Serrano, M., & Kroemer, G. (2013). The hallmarks of aging. Cell, 153(6), 1194–1217. 10.1016/j.cell.2013.05.039, 23746838 PMC3836174

[bib64] Mackenzie, C. (2000). Adult spoken discourse: The influences of age and education. International Journal of Language & Communication Disorders, 35(2), 269–285. 10.1080/136828200247188, 10912255

[bib65] Madden, D. J. (1988). Adult age differences in the effects of sentence context and stimulus degradation during visual word recognition. Psychology and Aging, 3(2), 167–172. 10.1037/0882-7974.3.2.167, 3268255

[bib66] Markiewicz, R., Rahman, F., Fernandes, E. G., Limachya, R., Wetterlin, A., Wheeldon, L., & Segaert, K. (2025). Effects of healthy ageing and bilingualism on attention networks. Bilingualism: Language and Cognition, 28(3), 802–815. 10.1017/S1366728924000154

[bib67] Matchin, W. (2023, October 24–26). Brain age predicts sentence processing declines in healthy aging beyond chronological age and domain general working memory abilities [Poster presentation]. Society for the Neurobiology of Language 15th Annual Meeting, Marseille, France.

[bib68] Mooraj, Z., Salami, A., Campbell, K. L., Dahl, M. J., Kosciessa, J. Q., Nassar, M. R., Werkle-Bergner, M., Craik, F. I. M., Lindenberger, U., Mayr, U., Rajah, M. N., Raz, N., Nyberg, L., & Garrett, D. D. (2025). Toward a functional future for the cognitive neuroscience of human aging. Neuron, 113(1), 154–183. 10.1016/j.neuron.2024.12.008, 39788085 PMC13032885

[bib69] Nasreddine, Z. S., Phillips, N. A., Bédirian, V., Charbonneau, S., Whitehead, V., Collin, I., Cummings, J. L., & Chertkow, H. (2005). The Montreal Cognitive Assessment, MoCA: A brief screening tool for mild cognitive impairment. Journal of the American Geriatrics Society, 53(4), 695–699. 10.1111/j.1532-5415.2005.53221.x, 15817019

[bib70] Nyberg, L., Lövdén, M., Riklund, K., Lindenberger, U., & Bäckman, L. (2012). Memory aging and brain maintenance. Trends in Cognitive Sciences, 16(5), 292–305. 10.1016/j.tics.2012.04.005, 22542563

[bib71] Obler, L. K., Fein, D., Nicholas, M., & Albert, M. L. (1991). Auditory comprehension and aging: Decline in syntactic processing. Applied Psycholinguistics, 12(4), 433–452. 10.1017/S0142716400005865

[bib72] Oschwald, J., Guye, S., Liem, F., Rast, P., Willis, S., Röcke, C., Jäncke, L., Martin, M., & Mérillat, S. (2019). Brain structure and cognitive ability in healthy aging: A review on longitudinal correlated change. Reviews in the Neurosciences, 31(1), 1–57. 10.1515/revneuro-2018-0096, 31194693 PMC8572130

[bib73] Peelle, J. E. (2019). Language and aging. In G. I. de Zubicaray & N. O. Schiller (Eds.), The Oxford handbook of neurolinguistics (pp. 295–316). Oxford University Press. 10.1093/oxfordhb/9780190672027.013.12

[bib74] Pelletier, A., Bernard, C., Dilharreguy, B., Helmer, C., Le Goff, M., Chanraud, S., Dartigues, J.-F., Allard, M., Amieva, H., & Catheline, G. (2017). Patterns of brain atrophy associated with episodic memory and semantic fluency decline in aging. Aging, 9(3), 741–752. 10.18632/aging.101186, 28278492 PMC5391228

[bib75] Pichora-Fuller, M. K., Schneider, B. A., & Daneman, M. (1995). How young and old adults listen to and remember speech in noise. Journal of the Acoustical Society of America, 97(1), 593–608. 10.1121/1.412282, 7860836

[bib76] Rahman, F., Tsvetanov, K. A., Feron, J., Mullinger, K., Joyce, K., Gilani, A., Fernandes, E. G., Wetterlin, A., Wheeldon, L., Lucas, S. J. E., & Segaert, K. (2025). Explaining tip-of-the-tongue experiences in older adults: The role of brain-based and cardiorespiratory fitness factors. Neurobiology of Aging, 154, 25–36. 10.1016/j.neurobiolaging.2025.06.008, 40582244

[bib77] Raz, N., Ghisletta, P., Rodrigue, K. M., Kennedy, K. M., & Lindenberger, U. (2010). Trajectories of brain aging in middle-aged and older adults: Regional and individual differences. NeuroImage, 51(2), 501–511. 10.1016/j.neuroimage.2010.03.020, 20298790 PMC2879584

[bib78] Raz, N., Lindenberger, U., Rodrigue, K. M., Kennedy, K. M., Head, D., Williamson, A., Dahle, C., Gerstorf, D., & Acker, J. D. (2005). Regional brain changes in aging healthy adults: General trends, individual differences and modifiers. Cerebral Cortex, 15(11), 1676–1689. 10.1093/cercor/bhi044, 15703252

[bib79] Rizio, A. A., & Diaz, M. T. (2016). Language, aging, and cognition: Frontal aslant tract and superior longitudinal fasciculus contribute toward working memory performance in older adults. NeuroReport, 27(9), 689–693. 10.1097/WNR.0000000000000597, 27138951 PMC4955947

[bib80] Rossi, E., & Diaz, M. T. (2016). How aging and bilingualism influence language processing: Theoretical and neural models. Linguistic Approaches to Bilingualism, 6(1–2), 9–42. 10.1075/lab.14029.ros, 28919933 PMC5600288

[bib81] Rothman, J., Bayram, F., DeLuca, V., Di Pisa, G., Duñabeitia, J. A., Gharibi, K., Hao, J., Kolb, N., Kubota, M., Kupisch, T., Laméris, T., Luque, A., van Osch, B., Periera Soares, S. M., Prystauka, Y., Tat, D., Tomić, A., Voits, T., & Wulff, S. (2023). Monolingual comparative normativity in bilingualism research is out of “*control*”: Arguments and alternatives. Applied Psycholinguistics, 44(3), 316–329. 10.1017/S0142716422000315

[bib82] Salthouse, T. A., & Mandell, A. R. (2013). Do age-related increases in tip-of-the-tongue experiences signify episodic memory impairments? Psychological Science, 24(12), 2489–2497. 10.1177/0956797613495881, 24104505 PMC4291522

[bib83] Schad, D. J., Nicenboim, B., Bürkner, P.-C., Betancourt, M., & Vasishth, S. (2023). Workflow techniques for the robust use of Bayes factors. Psychological Methods, 28(6), 1404–1426. 10.1037/met0000472, 35266787

[bib84] Segaert, K., Lucas, S. J. E., Burley, C. V., Segaert, P., Milner, A. E., Ryan, M., & Wheeldon, L. (2018). Higher physical fitness levels are associated with less language decline in healthy ageing. Scientific Reports, 8(1), Article 6715. 10.1038/s41598-018-24972-1, 29712942 PMC5928071

[bib85] Shafto, M. A., Burke, D. M., Stamatakis, E. A., Tam, P. P., & Tyler, L. K. (2007). On the tip-of-the-tongue: Neural correlates of increased word-finding failures in normal aging. Journal of Cognitive Neuroscience, 19(12), 2060–2070. 10.1162/jocn.2007.19.12.2060, 17892392 PMC2373253

[bib86] Sommers, M. S., & Danielson, S. M. (1999). Inhibitory processes and spoken word recognition in young and older adults: The interaction of lexical competition and semantic context. Psychology and Aging, 14(3), 458–472. 10.1037/0882-7974.14.3.458, 10509700

[bib87] Stamatakis, E. A., Shafto, M. A., Williams, G., Tam, P., & Tyler, L. K. (2011). White matter changes and word finding failures with increasing age. PLOS One, 6(1), Article e14496. 10.1371/journal.pone.0014496, 21249127 PMC3017545

[bib88] Steen-Baker, A. A., Ng, S., Payne, B. R., Anderson, C. J., Federmeier, K. D., & Stine-Morrow, E. A. L. (2017). The effects of context on processing words during sentence reading among adults varying in age and literacy skill. Psychology and Aging, 32(5), 460–472. 10.1037/pag0000184, 28816473

[bib89] Steffener, J., Habeck, C., O’Shea, D., Razlighi, Q., Bherer, L., & Stern, Y. (2016). Differences between chronological and brain age are related to education and self-reported physical activity. Neurobiology of Aging, 40, 138–144. 10.1016/j.neurobiolaging.2016.01.014, 26973113 PMC4792330

[bib90] Stine-Morrow, E. A. L., Soederberg Miller, L. M., Gagne, D. D., & Hertzog, C. (2008). Self-regulated reading in adulthood. Psychology and Aging, 23(1), 131–153. 10.1037/0882-7974.23.1.131, 18361662 PMC2577171

[bib92] Tucker, A. M., & Stern, Y. (2011). Cognitive reserve in aging. Current Alzheimer Research, 8(4), 354–360. 10.2174/156720511795745320, 21222591 PMC3135666

[bib91] Tucker-Drob, E. M. (2011). Global and domain-specific changes in cognition throughout adulthood. Developmental Psychology, 47(2), 331–343. 10.1037/a0021361, 21244145 PMC5374863

[bib93] Tyler, L. K., Shafto, M. A., Randall, B., Wright, P., Marslen-Wilson, W. D., & Stamatakis, E. A. (2010). Preserving syntactic processing across the adult life span: The modulation of the frontotemporal language system in the context of age-related atrophy. Cerebral Cortex, 20(2), 352–364. 10.1093/cercor/bhp105, 19505991 PMC2803734

[bib94] Wagenmakers, E.-J., Lodewyckx, T., Kuriyal, H., & Grasman, R. (2010). Bayesian hypothesis testing for psychologists: A tutorial on the Savage–Dickey method. Cognitive Psychology, 60(3), 158–189. 10.1016/j.cogpsych.2009.12.001, 20064637

[bib95] Wagenmakers, E.-J., Marsman, M., Jamil, T., Ly, A., Verhagen, J., Love, J., Selker, R., Gronoau, Q. F., Šmíra, M., Epskamp, S., Matzke, D., Rouder, J. N., & Morey, R. D. (2018). Bayesian inference for psychology. Part I: Theoretical advantages and practical ramifications. Psychonomic Bulletin & Review, 25(1), 35–57. 10.3758/s13423-017-1343-3, 28779455 PMC5862936

[bib96] Waters, G. S., & Caplan, D. (2001). Age, working memory, and on-line syntactic processing in sentence comprehension. Psychology and Aging, 16(1), 128–144. 10.1037/0882-7974.16.1.128, 11302362

[bib97] West, J. F., Sands, E. S., & Ross-Swain, D. (1998). Bedside evaluation screening test (BEST-2) (2nd ed.). Pro-Ed.

[bib98] Wittens, M. M. J., Denissen, S., Sima, D. M., Fransen, E., Niemantsverdriet, E., Bastin, C., Benoit, F., Bergmans, B., Bier, J.-C., de Deyn, P. P., Deryck, O., Hanseeuw, B., Ivanoiu, A., Picard, G., Ribbens, A., Salmon, E., Segers, K., Sieben, A., Struyfs, H., … Engelborghs, S. (2024). Brain age as a biomarker for pathological versus healthy ageing—A REMEMBER study. Alzheimer’s Research & Therapy, 16(1), Article 128. 10.1186/s13195-024-01491-y, 38877568 PMC11179390

[bib99] Zahodne, L. B., Stern, Y., & Manly, J. J. (2015). Differing effects of education on cognitive decline in diverse elders with low versus high educational attainment. Neuropsychology, 29(4), 649–657. 10.1037/neu0000141, 25222199 PMC4362867

[bib100] Zhang, H., Sachdev, P. S., Wen, W., Kochan, N. A., Crawford, J. D., Brodaty, H., Slavin, M. J., Reppermund, S., Kang, K., & Trollor, J. N. (2013). Grey matter correlates of three language tests in non-demented older adults. PLOS One, 8(11), Article e80215. 10.1371/journal.pone.0080215, 24224044 PMC3818244

[bib101] Zhu, Z., Deng, J., Li, M., Qin, Y., Li, J., & Yang, Y. (2022). Processing speed mediates the relationship between brain structure and semantic fluency in aging. Neuroscience Letters, 788, Article 136838. 10.1016/j.neulet.2022.136838, 35964825

